# Reduction of Dislocation of the Thumb

**Published:** 1855-12

**Authors:** 


Reduction of Dislocation of the Thumb.—M. Verhaeghe describes a plan he first employed upon the recommendation of Langenbeck, who had repeatedly found it successful. When the patient has been chloroformized the surgeon grasps with one hand the dislocated thumb, and carries it back, so to say, almost to the dorsal surface of the metacarpal bone. At the same time, with the point of the index finger of the other hand he presses up the head of the metacarpal bone, and with his thumb bears down the luxated extremity of the first phalanx. After the reduction the thumb should be kept fixed awhile by the starch bandage, or the dislocation will recur.—*Rev. Medico-Chir.*

